# Development and Acceptability of a Dietitian Delivered Patient Educational Video for Managing Irritable Bowel Syndrome

**DOI:** 10.1177/15598276261472814

**Published:** 2026-07-28

**Authors:** Jennifer Armstrong, Shannon Morley, Jennifer Utter

**Affiliations:** 1 Dietetics Department, 7392 Mater Health, South Brisbane, QLD, Australia (JA), (SM), (JU); 2 Nutrition and Dietetics, 3555 Bond University, Robina, QLD, Australia (JU)

**Keywords:** irritable bowel syndrome, diet & lifestyle interventions, video education, technology in healthcare

## Abstract

**Purpose:** Irritable bowel syndrome is a common disorder of the gut-brain interaction, with guidelines advising lifestyle modification as first-line treatment. This contributes to high demand for dietetic services. As technological interventions may improve efficiency of care and patient experience, we aimed to develop a first-line patient educational video for irritable bowel syndrome. **Setting:** This project was set at Mater Health, Brisbane, Australia, where referrals outweigh dietetic capacity, resulting in prolonged patient wait times. **Intervention:** Video content was based on best-practice guidelines and created via collaboration between dietetics and the gastroenterology multidisciplinary team. **Outcomes:** Four short educational videos were created. Consumer and clinician feedback described the videos as educational and easy to understand. Clinicians reported they would recommend the videos to their patients. All consumers said they would recommend the videos to family/friends with irritable bowel syndrome and 88% said they would make dietary changes. After watching the videos, 45% felt they would not require a face-to-face dietetic appointment. **Lessons Learned:** Our project suggests that using video technology is feasible and acceptable to both consumers and clinicians for IBS education. These results have wider implications for video education to be adapted by other healthcare professionals across a range of chronic conditions.

“Video-based solutions enhance accessibility, particularly for rural or remote patients who may otherwise face logistical challenges in attending in-person appointments.”

## Background

Irritable bowel syndrome (IBS) is a chronic, relapsing disorder of the gut-brain interaction, with symptoms that can include abdominal pain, altered bowel motions and abdominal bloating and/or distension.^1^ IBS affects approximately 5-10% of the population globally^2^ and the prevalence of disorders of gut-brain interactions in Australia is similar to that of 25 other high income countries.^3^ IBS significantly affects a person’s overall health and functioning; people with IBS report worse health related quality-of-life than those with other chronic conditions, including diabetes and end-stage renal disease.^4^ Diet and lifestyle modifications are commonly endorsed as first-line treatment in the management of IBS.^5^ Examples of effective dietary and lifestyle modifications include eating regular meals, ensuring adequate fluid intake, fiber modification and reducing intakes of artificial sweeteners, carbonated drinks, and caffeine.^5–7^

Given the prevalence of IBS and the role of diet in symptom management, dietitians are exploring more efficient models of care for delivering IBS education and therapy^8,9^ and technology offers a unique opportunity to deliver nutrition education efficiently for this population. A systematic review of sixteen studies found that using technology to provide virtual education to patients with chronic conditions, such as diabetes, chronic obstructive pulmonary disease, and IBS was comparable, or more effective, than usual care.^10^ Additional research suggests that up to 75% of the population now seek health information online, and convenient access is becoming an expectation.^11^ Clinicians have tested the feasibility, acceptability, and cost efficiency of dietitians using pre-recorded webinars to deliver first-line education to IBS patients.^12,13^ However, webinars can be time intensive for patients, which may reduce their engagement with the material, as evidence suggests shorter educational videos are more engaging and likely to be more effective.^14,15^

Taking these factors into account, the current project describes the development of an education video to provide first-line dietary advice to patients with irritable bowel syndrome who are on a wait-list to see a dietitian at Mater South Brisbane and to assess patient and clinician acceptability of this video.

## Setting and Practice Problem

The current project was undertaken at Mater Hospital South Brisbane. Mater Health South Brisbane is a large (approximately 800 beds), urban, tertiary healthcare campus delivering dietetic services both in hospital and in outpatient settings. In the outpatient setting, the number of patients referred for IBS symptom management greatly outweighs dietetic capacity. As a result, waiting times are long with some patients waiting up to a year for an initial dietetic appointment.

An internal audit found that IBS referrals make up approximately 50% of total outpatient gastroenterology dietetic referrals. This means that patients referred for IBS advice are often triaged as a category 3 referral and are seen within 365 days (or sooner if capacity allows). The audit also revealed very high failure to attend rates (>65%) at the first appointment. The audit findings prompted a review of this model of care, with the view to develop and implement a first-line educational video to be sent out at point of referral.

## Intervention

### Development of the Patient Education Video

The video content creation was led by a senior dietitian with extensive clinical expertise in gastroenterology and supported by a multidisciplinary team of gastroenterologists, specialist nurses and psychologists and dietitians. First, existing scientific literature was reviewed, along with a review of current clinical best-practice guidelines, to identify the key nutrition and behavioral messages to include in the video. The team agreed that the video content would prioritize diet and lifestyle modification and exclude low FODMAP (fermentable oligosaccharides, disaccharides, monosaccharides and polyols) dietary advice as low FODMAP advice requires more in-depth dietetic support. As a result, the video content included the following nutrition and lifestyle topics: eating regular meals, techniques for stress reduction, practicing mindful eating, ensuring adequate fluid intake, fiber modification and strategies for reducing intakes of caffeine, alcohol and artificial sweeteners (Table 1). To optimize patient engagement and maintain clarity of key dietetic messages, the goal was to limit the video length to 15 minutes.

**Table 1.** Content and Key Behaviors Targeted in Patient Education Videos for Managing Symptoms of Irritable Bowel Syndrome (IBS).

​	Topics covered	Key behaviors targeted
1 Understanding IBS	Symptoms Optimal stool consistency and frequency	​
2 Dietary advice for IBS management	Role of meal skipping and poor diet in IBS symptoms Relationship of stress and symptom onset/severity Symptom patterns associated with caffeine, alcohol, fatty foods and carbonated beverages	Eating 3 meals a day, prioritizing a healthy breakfast Avoid skipping meals Practice mindful eating (e.g., eat slowly) Avoiding or limiting coffee, alcoholic drinks and carbonated soft drinks Choosing healthier snacks
3 Fiber modification, including supplementation	Defining soluble fiber, insoluble fiber, and resistant starch Benefits of fiber (by type) for IBS Options for fiber supplements	Aim for 25-30g fiber per day, increase slowly Aim for 2-3L fluids per day Increasing consumption of fiber-rich foods, such as beans and pulses
4 Managing symptom patterns for IBS	Describing common symptom patterns experienced by people with IBS Role of prebiotics and probiotics in IBS	Explore other lifestyle behaviors for managing symptoms such as being physically active, gut-directed hypnotherapy and relaxation.

The in-house marketing team at Mater filmed and edited the video. Visual animations, diagrams and bullet points of key messages were added to enhance engagement and clarity of messages. The initial draft video was 13 minutes long. The Mater Human Research Ethics Committee reviewed the protocol for the current project and confirmed it met the criteria for a quality improvement project (not human subjects research) and, as such, means it is exempt from full review by the Committee (QACR/MML/120459).

### Assessing Clinician and Patient Acceptability

Clinicians working in gastroenterology and patient consumers were asked to review the video and complete a short questionnaire about it. Participation in reviewing the video was voluntary and anonymous. For recruitment of clinicians, a standardized email was sent to all clinicians actively working in the Mater gastroenterology team. Recruitment of patient consumers was conducted via the Mater Consumers in Care Committee and by offering any patient admitted to hospital under gastroenterology the option to voluntarily watch the video and complete the short questionnaire. These invitations happened over a four-week period and feedback on the video was returned anonymously. Ultimately, ten clinicians and ten patients reviewed the video and provided feedback.

Clinicians were asked to comment on the appropriateness of terminology used, length of the video, and understandability of the key messages. Overall, feedback was very positive and the key messages were easy to understand. 60% of clinicians felt the video length was acceptable, while others reported the length may be too long and impact on patient engagement. Only a few minor changes to specific terminology were advised by clinicians. Furthermore, clinicians advised that they would be happy to recommend the video to their patients and would like to have the video playing in the gastroenterology clinic waiting areas.

Patient consumers were asked to watch the video and complete a short questionnaire asking about video length, wording used, take-home messages and practicality of advice given. More than 75% of patient consumers felt that the video length was acceptable and 100% felt the language used was appropriate. All patients surveyed said they would recommend the video to family or friends with IBS and 88% said they would make some of the dietary changes suggested in the video. After watching the video 45% felt they would not require a face-to-face dietetic appointment. To ensure the content was easily understood, consumers were asked to list the top 3 key messages they took away from watching the video. Key themes consumers reported were the importance of fiber; limiting stress; the benefits of mindful eating and regular meals; and that diet and lifestyle modifications can help manage symptoms.

Last, 5 dietitians independently completed a Patient Education Materials Assessment Tool Audiovisual (PEMAT A/V). This validated tool assesses the understandability and actionability of patient education materials, ensuring they are suitable for patients with a range of health literacy levels.^16^ The average PEMAT A/V score for the IBS video was 100% for actionability and 93% for understandability, indicating an easy to understand and practical video.

### Final Revision of the Patient Education Video

Following comprehensive feedback from both consumers and clinicians, only minor revisions were made to the video prior to dissemination. The initial draft video was 13 minutes long and feedback highlighted concerns that the video was too long. In response, the video was divided into 4 shorter videos, each 3 to 5 minutes long. Additionally clear section headings and subtitles were added.

### Implementation and Evaluation of the Patient Education Video

The patient education videos are now available on the Mater YouTube channel, allowing patients and the community to access the resources at their convenience. Link to video 1: https://www.youtube.com/watch?v=wQQ_-_L2xdQ. Video 2: https://www.youtube.com/watch?v=exrBB1rkQTw. Video 3: https://www.youtube.com/watch?v=0JQ6Sq02YFE. Video 4: https://www.youtube.com/watch?v=qeEyAFR5svk. Clinicians within the organization have been encouraged to share the video with appropriate patients as part of routine clinical care. Additionally, the videos have been integrated into the dietetics model of care for patients with IBS and will be offered to publicly funded IBS patients prior to their initial dietetic appointment.

## Lessons Learned and Future Opportunities

This project has shown that video technology is feasible and acceptable to both consumers and clinicians at Mater South Brisbane in terms of IBS patient education. Positive feedback was provided from both consumers and clinicians with most consumers saying they would recommend the videos to family and friends with IBS and would make some of the dietary changes suggested in the videos. These results suggest that consumers at Mater are happy with video education as a first-line resource and that, in this study, approximately half felt that video education was sufficient to not require a face-to-face dietetic appointment. This has the potential to help relieve the demand on dietetic services at Mater, and in turn help reduce clinic waiting times. Ongoing evaluation will continue with a review of patient and clinician feedback, monitoring engagement metrics and assessment of the videos’ continued alignment with evolving clinical guidelines and patient needs. Future audits of the dietetic gastroenterology outpatient clinic will assess impact on patient experience, outcomes, and the efficiency of the model of care.

As these videos are now publicly available, they can also be assessed by patients and clinicians in the community. These results have wider implications for video education to be adapted by other healthcare professionals across a range of chronic diseases. When designed well, using evidence-based advice and focusing on patient-centered care, video education has the potential to enhance self-efficacy, improve clinical outcomes, and support a more sustainable healthcare delivery service.

With growing global demand for healthcare services, alongside limited capacity and funding to provide timely access, technology offers a viable solution. For conditions such as IBS, initial educational advice can be delivered via videos, reducing the need for immediate face-to-face consultations. Video education not only shortens the time to initial patient interaction but also allows patients to access the content at their convenience, revisit specific sections, and share the material with family or caregivers. Moreover, video-based solutions enhance accessibility, particularly for rural or remote patients who may otherwise face logistical challenges in attending in-person appointments.
